# Prediction of Retinopathy of Prematurity Using the WINROP (Weight, IGF-1, Neonatal Retinopathy of Prematurity) Algorithm in a South African Population

**DOI:** 10.3389/fped.2022.812404

**Published:** 2022-03-23

**Authors:** Samantha Jane Kesting, Firdose Lambey Nakwa

**Affiliations:** Department of Paediatrics, Division of Neonatology, Faculty of Health Sciences, School of Clinical Medicine, Chris Hani Baragwanath Academic Hospital, University of the Witwatersrand, Johannesburg, South Africa

**Keywords:** IGF-1, retinopathy, prematurity, WINROP, NICU, blindness

## Abstract

**Aim:**

This study aimed to assess the efficacy of the WINROP (Weight, IGF-1, Neonatal Retinopathy of Prematurity) screening algorithm in a South African population.

**Methods:**

A retrospective record review included infants born between 1 January 2013 and 1 December 2014 who underwent ROP (retinopathy of prematurity) screening. Outcomes of ophthalmology examinations were compared to alarms triggered on WINROP after gestational age, date of birth, and weekly weights were entered. Sensitivity, specificity, positive predictive, and negative predictive values and mean time of alarm were calculated.

**Results:**

Rates of ROP were 5.9% for all stages of ROP and 2.3% for severe ROP in the 220 infants included. Mean gestation age was 29.1 ± 1.3 weeks and mean birth weight 1,115.5 ± 201 g. WINROP triggered high-risk alarms in 70.5% of infants at a mean of 30.7 weeks of gestational age. Sensitivity for severe ROP was 100 and 76.9% for all stages of ROP. Specificity was low for both severe ROP and all stages of ROP at 30.2 and 30.0%, respectively.

**Conclusion:**

Rates of ROP are low in this population. The high number of alarms with a low negative predictive value would reduce the number of screens by 29.5%. Alarms were triggered before scheduled screening, possibly helpful in planning discharges and follow-up visits.

## Introduction

Retinopathy of prematurity (ROP) has been found to be the third leading cause of avoidable blindness worldwide (1). In developing countries where neonatal intensive care is still evolving, oxygen therapy is often liberally used and inadequately monitored, placing infants at risk of ROP (2). At the Chris Hani Baragwanath Academic Hospital, Mayet et al. found the mean birth weight of neonates with ROP to be 1,093.7 g, compared to those without ROP at 1,215.6 g. No severe ROP was found in infants above 1,250 g. Although ROP was found in 16.3% of infants, disease requiring treatment was found only in 1.6% (3).

The South African screening guideline recommends screening neonates born below 32 weeks of gestation, weighing <1,500 g at birth or with risk factors such as family history, cardiac arrest, multiple blood transfusions or exchange transfusions, and hypoxic ischemic encephalopathy. The examinations are to be conducted between 31 and 32 weeks of corrected gestational age or between 4 and 6 weeks of chronological age, whichever is later (4). At the Chris Hani Baragwanath Academic Hospital, studies have shown that gestational age is an unreliable factor to assess risk in our population due to infrequent first-trimester ultrasound scans and uncertain last menstrual periods. For this reason, the screening criteria are based on birth weight (3). A shortage of specialized ophthalmology services and screening programs resulted in only 19.2% of infants who fulfill the screening criteria being examined by an ophthalmologist in South Africa (5).

WINROP is a web-based computer program that works from a reference model calculated using logistic regression with expected values from the weights and IGF-1 levels of infants with no or mild ROP (6). Once the sex, gestational age at birth, and weekly weights have been entered, risk is indicated as red or green lamps indicating high or low risks, respectively. The timing of a high-risk alarm is also indicated in gestational age in weeks. The final ophthalmology examination is also inserted into the online database.

WINROP has been validated using retrospective cohort studies for the prediction of severe ROP in Europe, North America, South America, and Asia with sensitivities ranging from 84.7 to 100% (6–13). No data have been published regarding the efficacy of WINROP in an African population.

This study aimed to assess the efficacy of WINROP as a screening tool in predicting ROP in infants undergoing ROP screening at the Chris Hani Baragwanath Academic Hospital.

## Methods

A retrospective review of the records of patients who underwent ROP screening as per guidelines at the Chris Hani Baragwanath Academic Hospital was undertaken. Registration for WINROP was undertaken prior to commencement of the study with The Sahlgrenska Center for Pediatric Ophthalmology Research, allowing us access to the online program with a password-protected login. The study was approved by the University of the Witwatersrand Human Research Ethics Committee, clearance number M151123.

Weekly ROP screenings are performed by registrars in ophthalmology and referred to consultants as required. ROP was classified according to the International Classification of ROP (14). The ophthalmology reports of the routine ROP screenings were reviewed along with the patient records to obtain the required data. As only the inpatient records were available for review, only screenings done as an inpatient could be recorded. Not all patients had completed ROP screening by the time of discharge, and so the ROP diagnosis at the time of discharge was used for the study.

The gestational age, date of birth, weekly weights, and final ophthalmology screening results were entered into the online WINROP algorithm. Patient identifiers such as names and hospital numbers were recorded in a separate document to ensure confidentiality. The ETROP study (15) was the basis for the categorization in the study such as no ROP, mild ROP, and severe ROP as done in a previous evaluation of WINROP for uniformity (6).

Patient records were reviewed for infants born between 1 January 2013 and 31 December 2014 who qualified for ophthalmology ROP screening. The screening guidelines for ROP as per the Chris Hani Baragwanath Academic Hospital guidelines have the following inclusion criteria:

1. All infants with a birth weight of below 1,500 g
2. Infants with a birth weight of 1,500–2,000 g who received mechanical ventilation for more than 7 days
3. Infants with a birth weight of 1,500–2,000 g who received supplemental oxygen for more than 2 weeks
4. Infants with any birth weight who received supplemental oxygen for more than 6 weeks.

**Figure 1:**
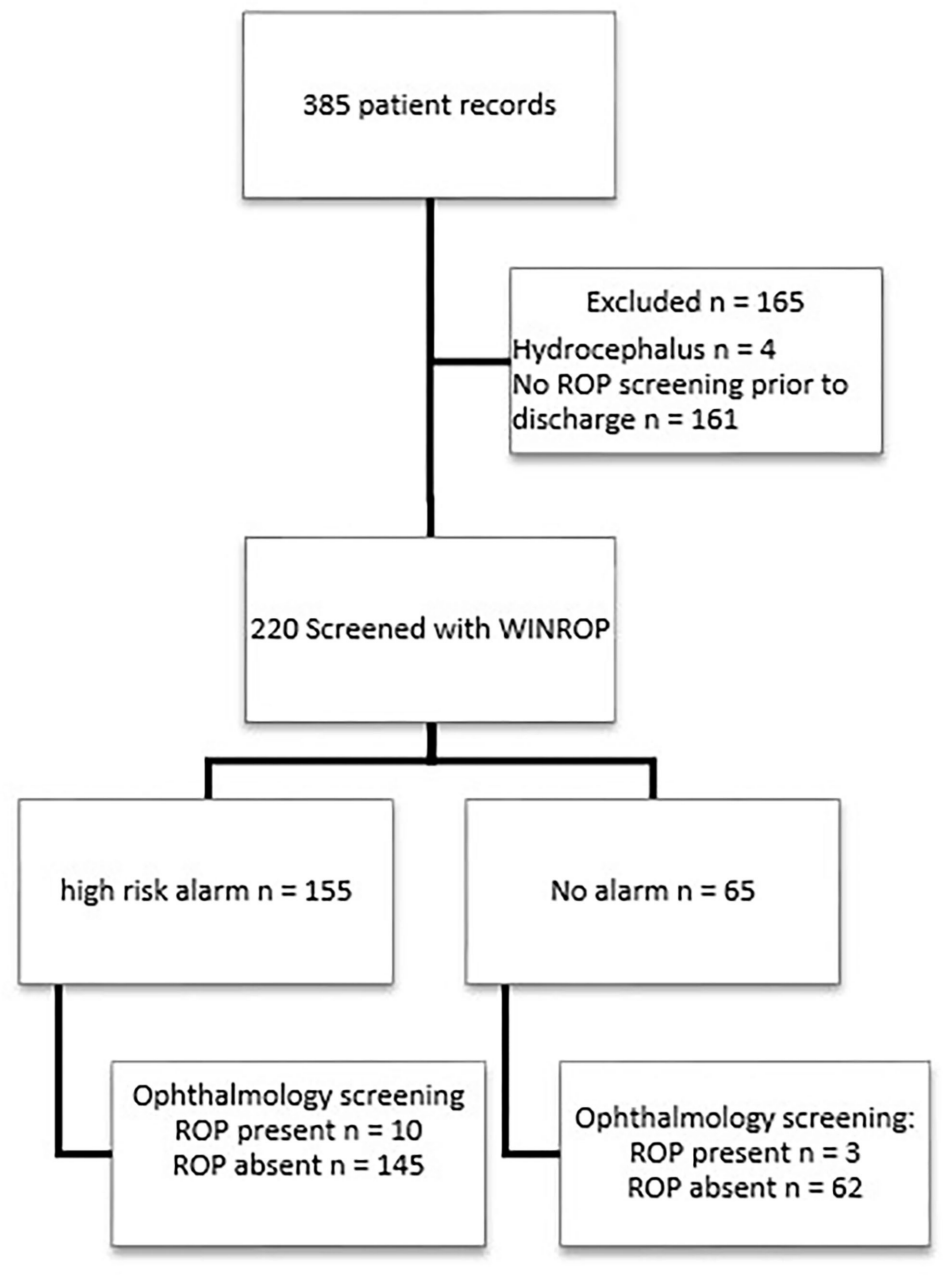
Flow of participants.

WINROP can be used reliably only for a gestational age at birth of 23 weeks + 0 days to 31 weeks + 6 days. For this reason, infants screened by the ophthalmology unit as per the guidelines listed above, but with a gestation >32 weeks were excluded. Due to the majority of the gestational ages in our study being calculated by examination with the New Ballard Score (16), which estimates gestation to full weeks, those at 32 weeks were included as 31 weeks + 6 days. Neonates that were noted to have conditions leading to disproportionate weights such as hydrocephalus were excluded (Figure 1).

The only other published study recording the prevalence of ROP in this setting was a study by Mayet which was published in 2006 (3). Using the frequency from this study of 16.3% for any ROP, a required sample size of 207 was calculated, allowing a margin for error of 5%, and a total of 220 patients were included.

The outcomes of the ophthalmology clinical examinations were then compared to the alarms triggered on the system. Sensitivity and specificity, as well as positive predictive and negative predictive values, were calculated based on high-risk alarms and clinical findings of ROP. The mean time of alarm and average weight gain per week were also calculated. The average weight gain per week was calculated using the difference between subsequent average weights per gestational and chronological age.

## Results

The rates of ROP in our population were low for all stages of ROP at 5.9% and severe ROP at 2.3%. Two patients were treated with bevacizumab injections by the ophthalmology unit as inpatients (Table 1). A total of 144 (97.3%) were of the African race.

**Table 1:** Patient characteristics and rates of ROP.

Number of infants	220
Gestational age mean (weeks)	29.1 ± 1.3
Birth weight mean (grams)	1.115 ± 201
Male gender [*n* (%)]	108 (49.1%)
No ROP [*n* (%)]	207 (94.1%)
ROP mild [*n* (%)]	8 (3.6%)
ROP severe [*n* (%)]	5 (2.3%)
ROP treated as inpatient [*n* (%)]	2 (0.9%)

The WINROP program triggered a high-risk alarm in 155 out of 220 infants (70.5%) at a mean of 30.7 ± 1.3 weeks of gestational age. Seventeen infants triggered an alarm at birth for being significantly small for gestational age, although none went on to develop any ROP (Table 2). All the severe-ROP patients triggered a high-risk alarm. Compared to the sensitivity for severe ROP (100%), the sensitivity for all stages of ROP was reduced to 76.9%. With the low specificity, the positive likelihood ratios were 1.1 for all stages of ROP and 1.4 for severe ROP.

**Table 2:** WINROP performance in predicting ROP.

	**Severe ROP**	**All ROP**
Sensitivity	100%	76.9%
Specificity	30.2%	30.0%
NPV	3.2%	6.5%
PPV	100%	95.4%
Positive likelihood ratio	1.4	1.1

## Discussion

The rates of ROP in our population were found to be low at 5.9% for all stages of ROP and 2.3% for severe ROP, when compared to other populations in the upper-middle-income countries (17). Despite the neonatal unit having far fewer oxygen blenders and less stringent oxygen saturation targets at the time, the rates of ROP were still low compared to other upper-middle-income countries with all stages of ROP at 16.3% and stage 3 of ROP at 2.5% (3). Another study in South Africa at Kalafong Provincial Tertiary Hospital by Delport et al. in 1999 (18) analyzed only the black infants screened and showed rates of 24.5% for all stages of ROP and 6.4% for stage 3 ROP. Other studies on ROP in South African populations have not commented on race for further comparison.

High-risk alarms were triggered by a large proportion of infants at the Chris Hani Baragwanath Academic Hospital (70%) considering the low rate of ROP (5.9%), resulting in a low negative predictive value. As a comparison, in other countries who have studied WINROP, Mexico had more alarms at 79.2% but also found severe ROP in 56.3% of their infants. In this population, ROP was found in larger, more mature infants. It was also disclosed that gestational age estimates, antenatal care, and oxygen monitoring were suboptimal (10).

The sensitivity of WINROP in our study was 100% for severe ROP but reduced for all stages of ROP at 76.9%. However, the positive likelihood ratios only indicate a minimal increase in the likelihood of disease with a high-risk alarm.

The high number of alarms appears to be related to our poor growth rates postnatally, which deviates from the algorithm used by WINROP. The average weight gain ranged from 0.66 to 12 g/day, which is far below the 15–17 g/kg/day recommended (19). Rooming facilities are limited in our setting, and expressed breast milk is not available in adequate volumes. Our donor breast milk bank is also limited and only available for a limited time to the smallest infants.

A study conducted in a multiracial London neonatal unit showed that black infants had lower levels of IGF-1 when compared to white patients. Along with the lower IGF-1 levels, they also had lower absolute postnatal weight gain. Despite these known risk factors, the black infants still needed less treatment for ROP than the white infants (20). The rates of severe ROP have also been found to be lower in infants of African descent in other studies in the USA, the United Kingdom, and Israel (21–24). This is suggested to be due to increased melanin, a known superoxide free radical in the retina. This would suggest that screening algorithms relying on growth and IGF-1 levels are population and race dependent, which may account for the differences in the efficacy of WINROP outside of Sweden, including in our study.

WINROP may assist in predicting those who are at highest risk of ROP requiring priority screening, although the low positive likelihood ratio only gives a minimal increase in the likelihood of disease. With such a high number of alarms, WINROP would only potentially reduce the numbers for screening by 30%. The alarm was triggered at a mean of 30.7 weeks of gestational age, which is before the routine screening by ophthalmology. This could perhaps assist in the planning of discharges and follow-up visits in infants discharged before the screening, as frequently happens in our hospital with a discharge weight of 1,650 g.

Rates of ROP were lower than the previous study in our population, which would influence the accuracy of the sample size calculation and therefore the power of the results. This was a prospective study and included all babies seen by ophthalmology for screening at birth during the period of July 2001 to December 2003 (3). It is likely that the reduction in the prevalence was due to the development of more stringent oxygen saturation protocols in the neonatal unit and the presence of more vital sign monitors and oxygen blenders than were available in 2003. These interventions are indeed not always present in a rural context, and so this would make it difficult to extrapolate to less-resourced units. In a survey of current oxygen management, screening criteria, and methods for treating ROP in sub-Saharan Africa, this unit is in a better-resourced position compared to the median of 3.5 oxygen measurement devices and 0 oxygen blenders and maximums of 70 and 50, respectively (25). These efforts in reducing the risk factors have resulted in a reduction in the rates of ROP in our population. However, due to resource constraints, infants below a weight of 1,000 g are not offered admission to the neonatal intensive care unit (NICU) and invasive ventilation, meaning the smallest premature infants are less likely to survive. The mean birth weights of infants from highly developed countries with severe ROP ranged from 737 to 763 g compared with from 903 to 1,527 g in less-developed countries (26).

As a retrospective record review of inpatient files, only the inpatient ROP screening examinations were captured. The final diagnosis or stage of ROP may have differed if the infants were referred or reviewed as outpatients to the ophthalmology department. With rotation of ophthalmology registrars through the department, there may be inconsistency in the screening examination. Gestational ages may be inaccurate as they are often based on last normal menstrual periods, symphysis pubis measurements, or postnatal New Ballard Scores performed by junior staff (16). The gestational age at birth is a critical part of the WINROP algorithm, influencing results.

## Conclusion

Rates of ROP are low at the Chris Hani Baragwanath Academic Hospital. This may be due to the vast majority of patients being African, which is known to be a protective factor against ROP.

WINROP showed 100% sensitivity but a low specificity and low negative predictive value secondary to a large proportion of high-risk alarms. The likelihood of disease was only minimally increased with a high-risk alarm. It appears that the test would be of limited benefit in our population. The increased alarms were due to poor postnatal weight gain that deviated from the algorithm. The poor growth in our infants is multifactorial and requires further investigation.

Our study seems to confirm that IGF-1 levels and growth are population and race dependent, resulting in differences in the performance of WINROP compared to those in other parts of the world. This could form the basis of a future prospective study. A future prospective study would be useful at a district or rural hospital where ophthalmology review is logistically difficult or not available as some benefit may be offered by reducing the number of referrals required.

## Data Availability Statement

The raw data supporting the conclusions of this article will be made available by the authors, without undue reservation.

## Ethics Statement

The studies involving human participants were reviewed and approved by University of the Witwatersrand Human Research Ethics Committee. Written informed consent from the participants' legal guardian/next of kin was not required to participate in this study in accordance with the national legislation and the institutional requirements.

## Author Contributions

SK and FN contributed to conception and design of the study and contributed to manuscript revision, read, and approved the submitted version. SK organized the database, performed the statistical analysis, and wrote the first draft of the manuscript. All authors contributed to the article and approved the submitted version.

## Funding

This work was supported by the Department of Paediatics, University of the Witwatersrand.

## Conflict of Interest

The authors declare that the research was conducted in the absence of any commercial or financial relationships that could be construed as a potential conflict of interest.

## Publisher's Note

All claims expressed in this article are solely those of the authors and do not necessarily represent those of their affiliated organizations, or those of the publisher, the editors and the reviewers. Any product that may be evaluated in this article, or claim that may be made by its manufacturer, is not guaranteed or endorsed by the publisher.

## Abbreviations

ETROP: Early treatment for retinopathy of prematurity
ROP: Retinopathy of prematurity
WINROP: Weight, IGF-1, Neonatal Retinopathy of Prematurity.
